# The Impact of Concomitant Diagnosis of Viral Infections on In-Hospital Mortality in Patients Hospitalized with a Diagnosis of Heart Failure in the United States: Insights from the National Inpatient Sample

**DOI:** 10.3390/v14112418

**Published:** 2022-10-31

**Authors:** Chun Shing Kwok, Kirellos Said Abbas, Adnan I. Qureshi, Duwarakan Satchithananda, Josip Andelo Borovac

**Affiliations:** 1 Department of Post-Qualifying Healthcare Practice, Birmingham City University, Birmingham B15 3TN, UK; 2 Department of Cardiology, University Hospitals of North Midlands NHS Trust, Stoke-on-Trent ST4 6QG, UK; 3 Faculty of Medicine, Alexandria University, Alexandria 21131, Egypt; 4 Zeenat Qureshi Stroke Institute and Department of Neurology, University of Missouri, Columbia, MO 65212, USA; 5 Clinic for Heart and Vascular Diseases, University Hospital of Split (KBC Split), 21000 Split, Croatia

**Keywords:** heart failure, viral infection, mortality, length of stay

## Abstract

The impact of viral infections on patients admitted with a diagnosis of heart failure is not well understood. We conducted a retrospective cohort study using data from the National Inpatient Sample in the United States to evaluate the proportion of admissions with a diagnosis of heart failure and viral infections, and we explored how viral infections had impact on in-hospital mortality and length of stay. There were a total of 20,713,539 admission records with a diagnosis of heart failure included in the analysis and 3.8% had a concomitant diagnosis of viral infection. The mean length of stay was 20.1 ± 26.9 days, 12.9 ± 13.6 days, 12.1 ± 13.8 days, and 5.1 ± 6.5 days for records with a diagnosis of cytomegalovirus, viral meningitis/encephalitis, herpes simplex infection, and no viral infection, respectively. The most common diagnoses of viral infections were influenza (*n* = 240,260) and chronic viral hepatitis (*n* = 194,400), and the highest rates of mortality were observed for records with a diagnosis of cytomegalovirus (13.2%), acute viral hepatitis (12.5%), and viral meningitis/encephalitis (11.1%). The viral infections significantly associated with increased odds of mortality were cytomegalovirus infection (OR 1.84 95% CI 1.57–2.16), acute hepatitis (OR 1.29 95% CI 1.15–1.45), and HIV (OR 1.22 95% CI 1.11–1.34). In conclusion, viral infections are co-diagnosis in 3.8% of patient records with heart failure and detection of some viruses may be important as they increase mortality and may prolong length of stay in hospital.

## 1. Introduction

Heart failure is a global problem responsible for considerable morbidity and mortality. In the developed world, the prevalence of known heart failure is generally estimated at 1% to 2% of the general adult population [1]. Quality of life in some patients with heart failure can be worse compared to many other chronic diseases, and mortality for hospitalized patients has been reported to be between 3% to 4% in the inpatient setting, which increases to 9% at 60 to 90 days, and over 20% at 1 year [2].

Infection represents an important emerging clinical problem that can cause decompensation in heart failure [3]. It has been reported that 25% of first hospitalizations in patients with heart failure with reduced ejection fraction were primarily due to infection [4]. While most viral infections of the heart are of little consequence, viral myocarditis can lead to cardiac damage, and severe acute heart failure can evolve progressively to chronic heart failure syndrome [5].

A few studies have investigated how viral infections impact mortality and length of stay in hospital admissions with heart failure from a national perspective. Influenza infections have been shown to be associated with increased in-hospital morbidity and mortality in heart failure patients [6]. Another analysis of the same national database suggests that there is no increase in mortality and length of stay with patients with human immunodeficiency virus (HIV) infection [7]. However, there are many other viral infections such as viral pneumonias, gastroenteritis, hepatitis and central nervous system conditions, together with specific viral infections, such as herpes simplex virus (HSV), herpes zoster virus (HZV), and cytomegalovirus (CMV), which are not well understood from a nationwide perspective. 

In this evaluation, we analyze nationally representative data from the United States to evaluate the proportion of admission records with a diagnosis of heart failure that also have a diagnosis of a viral infection. The primary outcome of the evaluation is in-hospital mortality for patients with and without specific infections.

## 2. Methods

This manuscript is prepared in accordance with the recommendations of the STROBE checklist [8]. Ethical approval was not required as we analyzed a non-identifiable dataset.

We analyzed nationally representative data in the United States from the National Inpatient Sample (NIS). The NIS is a database created by the Healthcare Cost and Utilization Project (HCUP) which is the largest publicly available all-payer inpatient healthcare database in the United States, which can be utilized to provide national estimates of inpatient utilization, access, costs, quality, and outcomes [9].

A retrospective cohort study was undertaken of all hospital records in the United States with a discharge diagnosis of heart failure between 2016 to 2019. These years were chosen because the hospital admission information was in ICD-9 codes prior to 2016. We excluded patients aged less than 18 years and missing values for death and sex. In order to produce national estimates for the analysis, the unweighted sample from the NIS was weighted by the discharge weights as recommended by the Healthcare Cost and Utilization project user support files [10].

From this group of hospital records, we defined a group of records with viral infections based on the individual diagnoses of viral infections defined by ICD-10 diagnostic codes and procedure codes as described in detail in Table A1. These viral infections included influenza, viral pneumonia, viral gastroenteritis, viral meningitis/encephalitis, HSV, HZV, acute viral hepatitis, chronic viral hepatitis, HIV, cytomegalovirus, infectious mononucleosis, viral conjunctivitis, adenovirus, enterovirus, parvovirus, and respiratory syncytial virus. Age was stratified according to whether patients were aged 18 to 60, >60 to 80 and >80 years of age. The discharge diagnosis codes, which were up to 40, were used to define coexisting illnesses and demographic, hospital information, and outcome data (in-hospital mortality, and length of stay) were available in the NIS dataset. The procedural codes, which were up to 25, were used to define the need for intubation and myocardial biopsy. The primary outcome was in-hospital mortality, and the secondary outcome was length of stay in hospital.

Statistical analysis was performed on Stata 13 (College Station, TX, USA). The hospital admissions were stratified by those which had any viral infection and no viral infection. Descriptive statistics were presented with both mean and standard deviation and median and interquartile range (IQR) for continuous variables and as percent for categorical variables. The non-parametric equality-of-medians test and student’s *t*-test on Stata were used to determine if there were any statistical differences for continuous variables and the Chi^2^ test was used for categorical variables. The frequency of the different individual diagnoses that made up any viral infection was determined together with the rate of mortality associated with each diagnosis. Both the median and mean length of stay for the individual diagnoses were presented. Mixed-effects multiple logistic regressions were used to identify independent predictors of in-hospital mortality with adjustments for demographic and comorbidity variables. The variables adjusted for were age, sex, race, smoking status, alcohol misuse, elective admission, weekend admission, season, year, primary expected payer, ZIP income quartile, hospital bed size, obesity, hypertension, hypercholesterolemia, diabetes mellitus, previous myocardial infarction, ischemic cardiomyopathy, dilated cardiomyopathy, myocarditis, atrial fibrillation, valvular heart disease, infective endocarditis, previous stroke, peripheral vascular disease, chronic kidney disease, liver failure, chronic lung disease, cancer, dementia, immunodeficiency, respiratory failure or arrest, dependence on ventilator, intubation, and myocardial biopsy. The variables in the model which evaluated in-hospital mortality for any viral infection vs. no viral infection were examined and the three variables most associated with in-hospital mortality were explored in a subgroup analysis to evaluate the influence of the variables on in-hospital mortality rates. A sensitivity analysis was performed evaluated the odds of in-hospital mortality in the group of patients aged >80 years.

## 3. Results

There were a total of 20,713,539 admission records with a diagnosis of heart failure included in the analysis (Appendix A—Figure A1). This included 779,250 records with any diagnosis of viral infection and 19,934,289 records with no diagnosis of viral infection. A total of 3.8% of this group of hospitalized records with a diagnosis of heart failure had a concomitant diagnosis of viral infection.

### 3.1. Baseline Characteristics and Comorbidities

The baseline characteristics and comorbidities of the records according to the presence or absences of a diagnosis of viral infection are shown in Table 1. The mean age of the patients was significantly younger for records with any diagnosis of viral infection compared to those without diagnosis of viral infection (68 ± 14 vs. 72 ± 14 years, *p* < 0.001). The proportion of female patients was 48.0% and 49.3% for records with any compared to no diagnosis of viral infection, respectively (*p* < 0.001). The proportion of patients who were white was lower among the records with a diagnosis of viral infection (60.8% vs. 70.1%, *p* < 0.001). There were proportionately fewer records with elective admission with any diagnosis of viral infections (4.8% vs. 9.7%, *p* < 0.001). The proportion of patients admitted in the winter was greater for records with any diagnosis of viral infection (39.6% vs. 25.5%). In terms of primary expected payer, records with any diagnosis of viral infections had a greater proportion with Medicaid (15.7% vs. 9.1%) but fewer patients with Medicare (69.7% vs. 75.7%). For comorbid illnesses, records with viral infections had proportionately fewer records with ischemic cardiomyopathy (7.9% vs. 10.5%, *p* < 0.001), obesity (19.1% vs. 22.5%, *p* < 0.001), hypercholesterolemia (44.5% vs. 52.6%, *p* < 0.001), diabetes mellitus (43.8% vs. 47.2%, *p* < 0.001), and atrial fibrillation (37.9% vs. 43.9%, *p* < 0.001). Patients admitted with a diagnosis of viral infections had greater proportions of myocarditis (0.2% vs. <0.1%, *p* < 0.001), chronic lung disease (46.6% vs. 40.4%, *p* < 0.001) and cancer (8.7% vs. 7.6%, *p* < 0.001). Immunodeficiency (0.5% vs. 0.2%, *p* < 0.001), respiratory failure or arrest (39.4% vs. 29.6%, *p* < 0.001), dependence on ventilator (0.7% vs. 0.5%, *p* < 0.001) and intubation (6.8% vs. 4.3%, *p* < 0.001) were more common in records with a diagnosis of viral infection. The in-hospital mortality was 5.3% for records with any viral infection and 4.7% for records with no viral infection (*p* < 0.001).

### 3.2. Length of Stay According to Diagnosis of Viral Infection

The mean and median lengths of hospital stay for admissions with a diagnosis of heart failure according to viral infections are shown in Table 2. The mean length of stay was greatest for records with cytomegalovirus (20.1 ± 26.9 days) and the length of stay for records with no viral infection was 6.1 ± 7.0 days. Other viral infections associated with long median length of stay were viral meningitis/encephalitis (12.9 ± 13.6 days), herpes simplex virus (12.1 ± 13.8 days), and infectious mononucleosis (11.9 ± 13.9 days). Records with viral gastroenteritis had the shortest length of stay (5.1 ± 6.5 days).

### 3.3. Number of Hospital Admission for Specific Viral Infections

**Figure 1:**
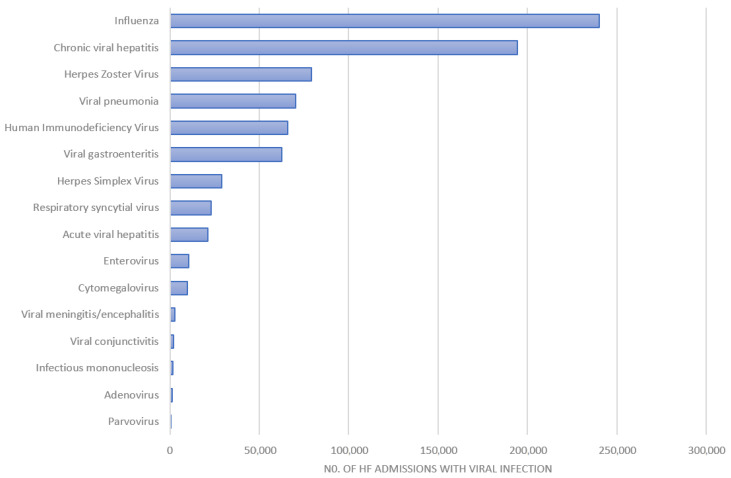
Admissions for viral infections among records with a diagnosis of heart failure.

The numbers of hospital admissions for patients with a diagnosis of heart failure and diagnoses of viral infections are shown in Figure 1. The most common diagnoses of viral infections were influenza (*n* = 240,260) and chronic viral hepatitis (*n* = 194,400), and influenza was present as a co-diagnosis in 1.2% of hospital admissions with a diagnosis of heart failure.

### 3.4. In-Hospital Mortality Rate and Adjusted Odds of Mortality for Specific Viral Infections

**Figure 2:**
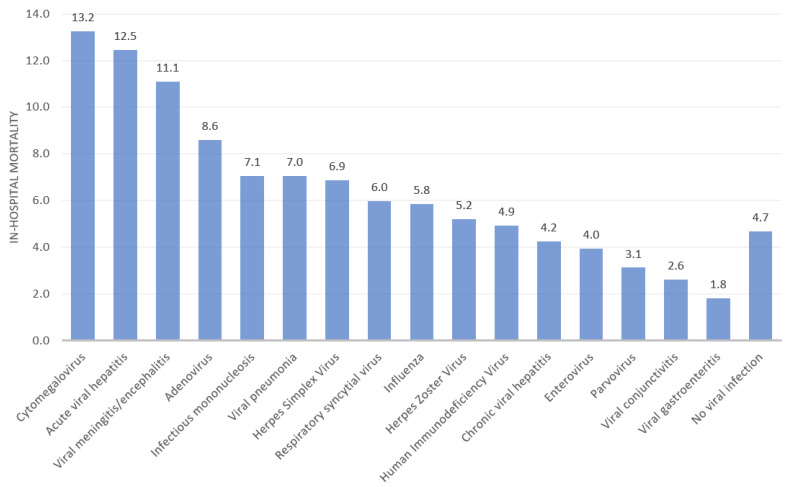
In-hospital mortality rate for records with a diagnosis of heart failure and viral infection.

The in-hospital mortality rates for records with viral infections and no viral infections with an admission with a diagnosis of heart failure are illustrated in Figure 2. The highest unadjusted rates of mortality were observed for records with a diagnosis of cytomegalovirus (13.2%), acute viral hepatitis (12.5%), and viral meningitis/encephalitis (11.1%), which was much greater than the rate for mortality for patients without a diagnosis of viral infection (4.7%). Mortality was low for records with co-existing diagnosis of viral conjunctivitis (2.6%) and viral gastroenteritis (1.8%).

**Figure 3:**
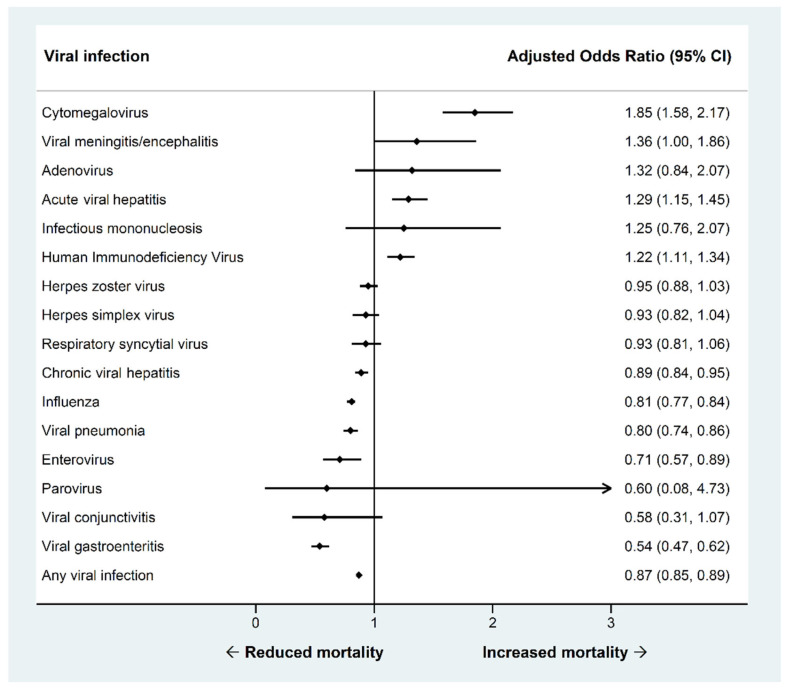
Adjusted odds of in-hospital mortality for records with a diagnosis of viral infection compared to those without a diagnosis of viral infection in patients admitted with heart failure.

The adjusted odds for in-hospital mortality for viral infection diagnoses compared to records without a diagnosis of viral infection are shown in Figure 3. The three viral infections most significantly associated with increased odds of mortality were cytomegalovirus infection (OR 1.85 95% CI 1.58–2.17), acute hepatitis (OR 1.29 95% CI 1.15–1.45), and HIV (OR 1.22 95% CI 1.11–1.34). There was significantly reduced odds of mortality compared to no infection for influenza (OR 0.81 95% CI 0.77–0.84), viral pneumonia (OR 0.80 95% CI 0.74–0.84), and viral gastroenteritis (OR 0.54 95% CI 0.47–0.62).

The variables in the model for any viral infection vs. no viral infection with the greatest odds of mortality were intubation (OR 7.34 95% CI 7.24–7.44, *p* < 0.001), liver failure (OR 5.19 95% CI 5.08–5.31, *p* < 0.001), and respiratory failure or arrest (OR 4.60 95% CI 4.54–4.65, *p* < 0.001). The impact of the intubation (34.0% vs. 3.4%), liver failure (28.4% vs. 4.3%), and respiratory failure or arrest (11.2% vs. 1.9%) variables had a much higher mortality rate compared to records that did not have the variables.

**Figure A2:**
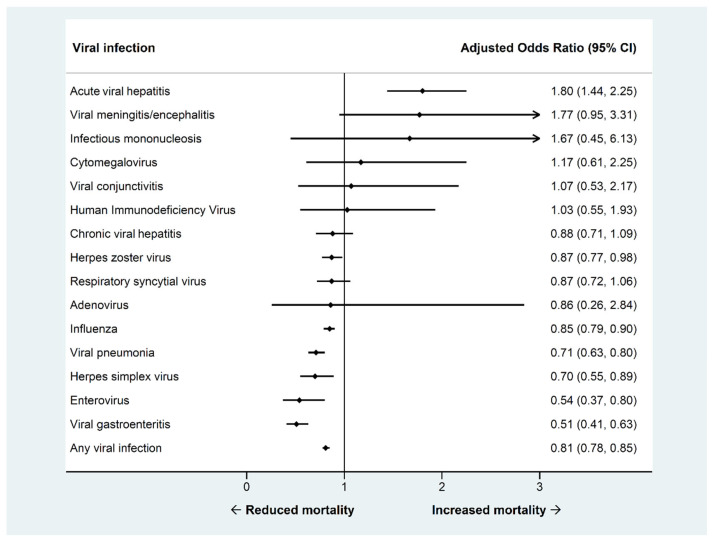
Adjusted odds of in-hospital mortality for patients with a diagnosis of viral infection compared to those without a diagnosis of viral infection in patients admitted with heart failure who are aged greater than 80 years.

When the cohort was restricted to patients who were over the age of 80 years, only acute viral hepatitis was associated with increased in-hospital mortality (Figure A2).

## 4. Discussion

This evaluation of patients hospitalized with a diagnosis of heart failure who also had a diagnosis of a viral infection has several key findings. First, viral infections are diagnosed in 3.8% of patients admitted with a diagnosis of heart failure, although this prevalence is likely higher given that a proportion of patient admitted due to HF might not be tested for the confirmation of viral illness. Secondly, patients with a diagnosis of viral infections were of lower age and more likely to present in the winter. These patients are also more likely to have chronic lung disease and respiratory failure or arrest. Third, in the hospitalized patients with heart failure, the most common viral infections were influenza and chronic viral hepatitis. Fourth, mortality rates vary depending on the type of viral infection, which can be more than 10% for cytomegalovirus infections, acute viral hepatitis, and viral meningitis/encephalitis, or as low as 2% for heart failure patients with viral conjunctivitis or viral gastroenteritis. Finally, length of stay is increased with some viral infections, particularly cytomegalovirus, viral meningitis/encephalitis, herpes simplex virus, and infectious mononucleosis. These findings suggest that patients with heart failure can present to hospital with viral infection and some, such as cytomegalovirus, acute viral hepatitis, and HIV, can be associated with increased in-hospital mortality, while length of stay may be greatly prolonged in patients with cytomegalovirus, viral meningitis/encephalitis, herpes simplex virus, and infectious mononucleosis.

We report some differences in findings from similar studies which focus on specific viral infections and heart failure. The previous national evaluation of influenza in heart failure found that influenza was independently associated with increased in-hospital mortality [6]. In the current evaluation, with more contemporary data and the use of ICD-10 codes, we found that once we adjusted for respiratory failure, dependence on a ventilator, and intubation, there was no significant increase in odds of in-hospital mortality. The other study of patients with HIV who were admitted with decompensated heart failure was only published in abstract form [7], so we are unable to ascertain the variables that were adjusted for in their analysis. This study reported a non-significant reduction in mortality and length of stay for patients with HIV. Our current evaluation differs from their study because we included all patients with heart failure, not just those who were diagnosed with acute decompensated heart failure, and that study only considered heart failure as the principal diagnosis when heart failure may not be the first diagnosis in the discharge records. The differences in methodological approaches between the current study and the previous study resulted in major differences in the number of patients admitted with a diagnosis of HIV that were captured, which was 975 in the other study compared to 66,040 in the current study.

An interesting finding of this study is the observation that a diagnosis of cytomegalovirus in hospitalized patients with heart failure is associated with significantly increased in-hospital mortality and longer length of stay in hospital compared to patients without a diagnosis of the infection. Cytomegalovirus is a widespread condition as approximately 59% of the population older than 6 years has been exposed to it and the seroprevalence increases with age [11]. There is a range of presentations associated with acute cytomegalovirus infection where patients are most commonly asymptomatic, but some can develop a mononucleosis syndrome, fever, pharyngitis, cervical adenopathy and non-specific rash, anemia, and more rarely, Guillain–Barre syndrome, encephalitis, myocarditis and pneumonitis [12]. A meta-analysis of 10 studies suggests that exposure to the infection was associated with a 22% increase in relative risk of future cardiovascular disease, and it was suggested in this review that 13.4% of cardiovascular disease may be attributed to cytomegalovirus [13]. In the context of heart failure, it may be important as a recent study suggests that active cytomegalovirus infection is uncommon in patients with acute heart failure but may be a marker of disease severity [14]. It is important to distinguish the potential that heart failure patients who are more ill are more likely to have tests and are thus found to have non-active cytomegalovirus infection as opposed to cytomegalovirus directly causing worsening of the clinical condition to contribute to increased mortality. In addition, cytomegalovirus is a virus which has been associated with fatal myocarditis [15]. Future studies are needed to better understand the association between cytomegalovirus and outcomes in heart failure as the testing for cytomegalovirus in the current study was at the discretion of the clinician, so there is no indication of which patients were tested and those who were not tested. 

The examination of variables associated with in-hospital mortality found that many of the patients who die in hospital have respiratory failure, intubation, and liver failure. It is not clear if respiratory failure and liver failure represent multiorgan failure in the context of viral infection or heart failure. Furthermore, it is uncertain what the causes for the respiratory and liver failure are. Respiratory failure may be a consequence of the progression of heart failure or the lung involvement in viral illness. The liver failure may be related to viral hepatitis, but there may also be other contributors, such as ischemic liver injury and medication-induced hepatic injury.

The approach of using national data was important because some infections are not commonly diagnosed in patients admitted to hospital with heart failure. Cytomegalovirus, acute hepatitis, and viral meningitis/encephalitis were not common diagnoses in the hospitalized cohort with heart failure in the current study as they were only present in 0.05%, 0.10%, and 0.01%, respectively. This may raise the possibility that studying the condition in smaller cohort may be challenging. However, it is also possible that there is undertesting so that these viral infections may be more common than expected. The challenge are viral infections where there are fewer obvious diagnostic features, such as cytomegalovirus, as many conditions such as acute hepatitis would likely be found when a patient has routine bloods that show abnormal liver function tests and viral meningitis/encephalitis may cause symptoms of fever, headache, vomiting, and confusion, which are not features of heart failure. Nevertheless, this study is important as this data from a national perspective identifies that this area of viral infections in heart failure merits further investigation.

Testing for viral infections when patients with heart failure present unwell is not part of recommended guidelines. In the current evaluation, the viral testing was at the discretion of the clinical team who looked after the patient, so we have no understanding of how many patients were tested and how many were not tested but might have benefited from testing. There are also patients that may have died before testing or frail or comorbid patients where decisions were made not to test and to care for patients palliatively. In addition, while there have been some improvements in the laboratory testing techniques to diagnose viral disease, there are limitations with the tests that are routinely used for viruses, such as the lack of availability of serological tests to identify the virus or antigen for all viruses, lack of sensitivity of diagnostic kits compared to polymerase chain reaction (PCR), and risk of detection of non-relevant co-infections or DNA contamination with detection of viral genomes by PCR [16]. However, if patients do not respond to the acute treatment of heart failure or patients present atypical symptoms, it may be worth considering investigating for infections. Misdiagnosis of heart failure has been reported in the literature and the most common cause for misdiagnosis was chronic obstructive pulmonary disease [17], and exacerbations of chronic obstructive pulmonary disease are frequently associated with respiratory virus infections [18]. Additionally, the challenge with viral infectious is that many do not cause symptoms, as one study suggests that asymptomatic infections rates exceed 70% for most viruses except influenza and human metapneumovirus [19]. Therefore, it is important that the virus has clinical implications, otherwise identification of whether the patient has the viral infection or has previously had the viral infection may not impact management or outcomes. It remains an outstanding question that merits further investigation: which patients who are admitted with heart failure should be screened for viral infections and which viral infections to test for. Identification of viral infections in some cases is important as some viral infections may be managed with antiviral medications, such as herpes simplex virus, cytomegalovirus, and HIV.

The findings of the current study, which are representative of hospital admissions in the United States, may not be generalizable to all other countries. The patterns and types of viral infections may differ according where the evaluation of viral infections take place. The United States is a large country with both urban and less populated areas, and tropical diseases may appear because of tourists and migration. Other factors may also impact viral infections such as the use of vaccines as the influenza vaccine has been shown to reduce the risk of influenza illness in the United States [20]. Vaccination for pneumococcal and influenza infection can improve outcomes and reduce severe outcomes among patients with HF [21]. Similarly, influenza and pneumococcal pneumonia were the most common vaccine-preventable infections among patients with HF with reduced ejection fraction, and these patients were at significantly greater risk of in-hospital mortality, length of stay, respiratory failure requiring mechanical ventilation, and other in-hospital complications [22].

In addition, several viral infectious have been shown to occur in immunocompromised hosts, which can cause severe morbidity and disseminated fatal infections if untreated, while many of these viruses are asymptomatic or self-limiting in immunocompetent hosts [23]. Therefore, rates of patients with viral infections in hospital settings may be affected by the population who are on immunosuppressive medication and patients with primary immunodeficiency and conditions such as type 1 diabetes mellitus and rheumatoid arthritis, which may vary depending on the country evaluated.

The evaluation has a few clinical implications. Investigating viral infections as an underlying cause for deterioration in patients with heart failure may be important. A recent retrospective analysis suggests that there is under reporting, testing and diagnosis of viral causes of admissions in heart failure patients [24]. This study also raises awareness of viral infections as a problem for patients with heart failure, and there may be benefits in preventing these types of infections where possible. A key preventative measure may be vaccination, which has been shown to be associated with a mortality among patients with heart failure [25].

This study has several limitations. First, the study is retrospective and observational, which places the findings at risk of potential confounding. Second, there is no understanding of the extent patients underwent viral infection testing or the diagnostic criteria for the diagnosis to be made. It is possible that some of the diagnoses were made based on clinical evaluation rather than viral testing. In addition, the exact onset of the viral syndrome is not known. This is important as if testing was not performed, then we cannot ascertain whether patients who did not have a diagnosis of viral infection did not have the diagnosis. Furthermore, there is no understanding of why the patients who had viral infection underwent testing and others did not. Third, the nature of the NIS is such as that the same patients may appear in the dataset multiple times within a year or across different years as there is no way of identifying individual patients. Fourth, there is no information about the management of the patients and the viral infection or the heart failure, which may have an impact on length of stay and mortality. Another important limitation is that the degree of systolic function as a continuous variable (left ventricular ejection fraction) was not available in the NIS; therefore, we might not ascertain the possible differential effect of viral infections on HF with reduced, mid-range, and preserved ejection fraction phenotypes. Another major limitation of this study is that we do not have the results of the biopsy. This is important as many of the viruses mentioned in the study are cardiotropic, so detection of the virus on myocardial biopsy is important. The consideration of the biopsy and its results is important for future work on heart failure and viral infections. In addition, it is possible that there are inaccuracies in the use of ICD-10 codes for heart failure and the variation in code selection has been reported across different NIS studies for heart failure [26]. However, in terms of accuracy, it has been reported that for surgery involving solid organ transplant, the codes are accurate and reliable for generating national estimates [27]. The current study is also limited because there is no information on the New York Heart Association classification or other marker of severity of the heart failure. Finally, this dataset was up to 2019 and we do not have data for the results from the year 2020 onwards, where COVID-19 was a problematic viral infection, so the results may not be generalizable to current clinical practice.

## 5. Conclusions

Viral infections are a co-diagnosis in many patients with heart failure. The most common viruses in the hospitalized patients with heart failure are influenza, chronic viral hepatitis, herpes zoster virus, and viral pneumonia. Detection of some viral infections may be important as cytomegalovirus, acute viral hepatitis, and HIV are associated with increased mortality, while others, such as viral meningitis/encephalitis, herpes simplex virus, and infectious mononucleosis, may prolong length of stay. More studies are needed to understand which patients should undergo virus testing and determine whether outcomes for patients can be improved with more thorough screening for viral infections and early vaccination for anyone at risk for heart failure admission.

## Figures and Tables

**Table 1:** Characteristics and comorbidities of records with and without viral infections with a hospital diagnosis of heart failure.

Variable	Admission without a Diagnosis of Viral Infection (*n* = 19,986,859)	Admission with a Diagnosis of Viral Infection (*n* = 155,850)	*p*-Value
Mean age in years (±SD)	72 ± 14	68 ± 14	<0.001
Median age in years [IQR]	73 [63 to 83]	68 [59 to 80]	<0.001
Age group 18 to 60 years >60 to 80 years >80 years	20.4% 48.4% 31.2%	29.4% 46.3% 24.3%	<0.001
Female sex	49.3%	48.0%	<0.001
Race White Black Hispanic Asian or Pacific Islander Native American Other	70.1% 17.4% 7.7% 2.0% 0.5% 2.2%	61.1% 24.0% 9.1% 2.7% 0.6% 2.5%	<0.001
Smoking	0.9%	1.0%	<0.001
Alcohol misuse	1.7%	2.8%	<0.001
Elective admission	9.7%	4.8%	<0.001
Weekend admission	22.7%	25.1%	<0.001
Season Spring Summer Fall Winter	25.8% 24.1% 24.6% 25.5%	28.2% 14.8% 17.4% 39.6%	<0.001
Year 2016 2017 2018 2019	23.0% 24.5% 25.6% 26.9%	17.9% 24.8% 29.8% 27.5%	<0.001
Primary expected payer Medicare Medicaid Private insurance Self-pay No charge Other	75.7% 9.1% 11.2% 2.0% 0.2% 1.8%	69.7% 15.7% 10.5% 2.2% 0.2% 1.7%	<0.001
ZIP income quartile 1st–25th 26th–50th 51st–75th 76th–100th	32.7% 26.8% 23.0% 17.5%	35.2% 24.9% 22.4% 17.5%	<0.001
Hospital bed size Small Medium Large	20.5% 29.5% 50.0%	18.9% 27.5% 53.5%	<0.001
Obesity	22.5%	19.1%	<0.001
Hypertension	88.1%	85.9%	<0.001
Hypercholesterolemia	52.6%	44.5%	<0.001
Diabetes mellitus	47.2%	43.8%	<0.001
Previous myocardial infarction	14.9%	12.9%	<0.001
Ischemic cardiomyopathy	10.5%	7.9%	<0.001
Dilated cardiomyopathy	3.4%	3.4%	0.74
Myocarditis	<0.1%	0.2%	<0.001
Atrial fibrillation	43.9%	37.9%	<0.001
Valvular heart disease	12.2%	9.8%	<0.001
Infective endocarditis	0.7%	1.1%	<0.001
Previous stroke	15.3%	13.4%	<0.001
Peripheral vascular disease	6.5%	5.1%	<0.001
Chronic kidney disease	44.5%	45.4%	<0.001
Liver failure	1.7%	4.0%	<0.001
Chronic lung disease	40.4%	46.6%	<0.001
Cancer	7.6%	8.7%	<0.001
Dementia	11.1%	9.3%	<0.001
Immunodeficiency	0.2%	0.5%	<0.001
Respiratory failure or arrest	29.6%	39.4%	<0.001
Dependence on ventilator	0.5%	0.7%	<0.001
Intubation	4.3%	6.8%	<0.001
Myocardial biopsy	0.1%	0.2%	<0.001
In-hospital mortality	4.7%	5.3%	<0.001
Mean length of stay (±SD)	6.1 ± 7.0	7.5 ± 9.5	<0.001
Median length of stay [IQR]	4 [3 to 7]	5 [3 to 9]	<0.001

IQR = interquartile range, SD = standard deviation.

**Table 2:** Mean and median length of stay for records with and without viral infections with a hospital diagnosis of heart failure.

Viral Infection	Mean Length of Stay in Days (±SD)	Median Length of Stay in Days [IQR]
Cytomegalovirus	20.1 ± 26.9	11 [6 to 24]
Viral meningitis/encephalitis	12.9 ± 13.6	9 [5 to 16]
Herpes simplex virus	12.1 ± 13.8	8 [4 to 15]
Infectious mononucleosis	11.9 ± 13.9	8 [4 to 13]
Parvovirus infection	10.1 ± 9.3	7 [3 to 12]
Adenovirus infection	10.1 ± 12.3	6 [3 to 12]
Acute viral hepatitis	9.2 ± 10.0	6 [3 to 11]
Viral pneumonia	8.6 ± 9.9	6 [4 to 10]
Herpes zoster infection	7.7 ± 9.7	5 [3 to 9]
Human immunodeficiency virus	7.7 ± 10.9	5 [3 to 9]
Viral conjunctivitis	7.3 ± 7.2	5 [3 to 9]
Chronic viral hepatitis	7.3 ± 9.3	5 [3 to 8]
Respiratory syncytial virus	7.1 ± 8.6	5 [3 to 9]
Enterovirus infection	7.1 ± 7.0	5 [3 to 8]
Influenza	7.0 ± 7.9	5 [3 to 8]
Viral gastroenteritis	5.1 ± 6.5	3 [2 to 6]
No viral infection	6.1 ± 7.0	4 [3 to 7]

IQR = interquartile range, SD = standard deviation.

**Figure A1:**
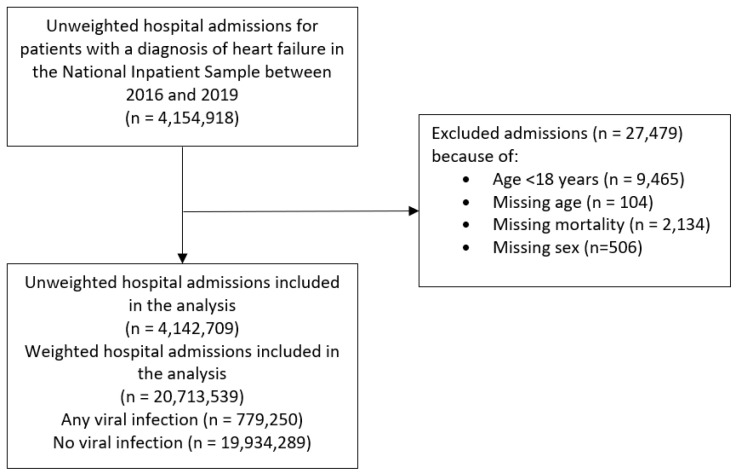
Flow diagram of hospital admissions with heart failure included in the analysis.

## Data Availability

The data used in this analysis may be purchased from the Healthcare Cost and Utilization Project website. The authors do not have permission to share the data used for the analysis.

## Appendix A

**Table A1:** Codes for data analysis and their source.

Variable	Source	ICD-10 Code
Influenza	I10_DX1/40	J09 *, J10 *, J11 *
Viral pneumonia	I10_DX1/40	J12 *
Viral gastroenteritis	I10_DX1/40	A08 *
Viral meningitis/encephalitis	I10_DX1/40	A83 *, A84 *, A85 *, A86 *, A87 *s
Herpes simplex infection	I10_DX1/40	B00 *
Herpes zoster infection	I10_DX1/40	B02 *
Acute viral hepatitis	I10_DX1/40	B15 *, B16 *, B17 *
Chronic viral hepatitis	I10_DX1/40	B18 *
Human immunodeficiency virus	I10_DX1/40	B20 *
Cytomegalovirus	I10_DX1/40	B25 *
Parvovirus	I10_DX1/40	B97.6
Adenovirus	I10_DX1/40	B97.0
Enterovirus	I10_DX1/40	B97.1
Respiratory syncytial virus	I10_DX1/40	B97.4
Infectious mononucleosis	I10_DX1/40	B27 *
Viral conjunctivitis	I10_DX1/40	B30 *
Any virus	-	Composite of all viral infections
Smoking (Tobacco use)	I10_DX1/40	Z72.0
Alcohol misuse	I10_DX1/40	F10.1
Obesity	I10_DX1/40	E66.0, E66.1, E66.2, E66.8, E66.9
Hypertension	I10_DX1/40	I10 *, I11 *, I12 *, I13 *, I15 *, I16 *
Hyperlipidemia	I10_DX1/40	E78.0 *, E78.1, E78.2, E78.3, E78.4 *, E78.5
Diabetes mellitus	I10_DX1/40	E08 *, E09 *, E10 *, E11 *, E13 *
Previous myocardial infarction	I10_DX1/40	I25.2
Ischemic cardiomyopathy	I10_DX1/40	I25.5
Dilated cardiomyopathy	I10_DX1/40	I42.0
Myocarditis	I10_DX1/40	I40 *, I41 *
Atrial fibrillation or flutter	I10_DX1/40	I48 *
Valvular heart disease	I10_DX1/40	I34 *, I35 *, I36 *, I37 *
Infective endocarditis	I10_DX1/40	I33 *, I38 *, I39 *
Previous stroke	I10_DX1/40	Z86.73, I69 *
Peripheral vascular disease	I10_DX1/40	I73 *
Chronic kidney disease	I10_DX1/40	N18 *
Liver failure	I10_DX1/40	K72 *
Chronic lung disease	I10_DX1/40	J40 *–J47 *
Cancer	I10_DX1/40	C00 *–C96 *
Dementia	I10_DX1/40	F01 *, F02 *, F03 *, G30 *, G31 *
Immunodeficiency	I10_DX1/40	D80 *, D81 *, D82 *, D83 *, D84 *
Respiratory failure or arrest	I10_DX1/40	J96 *, R09.2
Dependence on ventilator	I10_DX1/40	Z99.11
Intubation	I10_PR1/25	OBH17EZ
Myocardial biopsy	I10_PR1/25	02BL0ZX, 02BL3ZX, 02BL4ZX, 02BK0ZX, 02BK3ZX, 02BK4ZX, 02BM0ZX, 02BM3ZX, 02BM4ZX
Age	NIS Core	-
Sex	NIS Core	-
Month of admission	NIS Core	-
Weekend admission	NIS Core	-
Discharge weight	NIS Core	-
Discharge disposition	NIS Core	-
Elective admission	NIS Core	-
Length of stay	NIS Core	-
Primary expected payer	NIS Core	-
Race	NIS Core	-
Year	NIS Core	-
ZIP income quartile	NIS Core	-
Death	NIS Core	-
Hospital bed size	NIS Hospital	-

The * signifies truncation where the asterisk (*) represents any group of characters or no characters.
